# Traumatic Brain Injury: Oxidative Stress and Novel Anti-Oxidants Such as Mitoquinone and Edaravone

**DOI:** 10.3390/antiox9100943

**Published:** 2020-10-01

**Authors:** Helene Ismail, Zaynab Shakkour, Maha Tabet, Samar Abdelhady, Abir Kobaisi, Reem Abedi, Leila Nasrallah, Gianfranco Pintus, Yusra Al-Dhaheri, Stefania Mondello, Riyad El-Khoury, Ali H. Eid, Firas Kobeissy, Johnny Salameh

**Affiliations:** 1 Department of Neurology, American University of Beirut Medical Center, Beirut 1107 2020, Lebanon; hi47@aub.edu.lb; 2 Department of Biochemistry and Molecular Genetics, American University of Beirut, Beirut 1107 2020, Lebanon; zs69@aub.edu.lb (Z.S.); mt101@aub.edu.lb (M.T.); ak216@aub.edu.lb (A.K.); ra386@aub.edu.lb (R.A.); ln23@aub.edu.lb (L.N.); 3 Faculty of Medicine, Alexandria University, Alexandria 21544, Egypt; samar.abdelhady1244@alexmed.edu.eg; 4 Department of Medical Laboratory Sciences, University of Sharjah, Sharjah 27272, UAE; gpintus@sharjah.ac.ae; 5 Department of Biomedical Sciences, University of Sassari, 07100 Sassari, Italy; 6 Department of Biology, College of Science, United Arab Emirates University, Al-Ain 15551, UAE; yusra.aldhaheri@uaeu.ac.ae; 7 Department of Biomedical and Dental Sciences and Morphofunctional Imaging, University of Messina, 98165 Messina, Italy; stefania.mondello@unime.it; 8 Department of Pathology and Laboratory Medicine, Neuromuscular Diagnostic Laboratory, American University of Beirut Medical Center, Beirut 1107 2020, Lebanon; re70@aub.edu.lb; 9 Department of Pharmacology and Toxicology, American University of Beirut, Beirut 1107 2020, Lebanon; 10 Department of Biomedical Sciences, Qatar University, Doha 2713, Qatar

**Keywords:** traumatic brain injury, oxidative stress, anti-oxidants, edaravone, mitoquinone

## Abstract

Traumatic brain injury (TBI) is a major health concern worldwide and is classified based on severity into mild, moderate, and severe. The mechanical injury in TBI leads to a metabolic and ionic imbalance, which eventually leads to excessive production of reactive oxygen species (ROS) and a state of oxidative stress. To date, no drug has been approved by the food and drug administration (FDA) for the treatment of TBI. Nevertheless, it is thought that targeting the pathology mechanisms would alleviate the consequences of TBI. For that purpose, antioxidants have been considered as treatment options in TBI and were shown to have a neuroprotective effect. In this review, we will discuss oxidative stress in TBI, the history of antioxidant utilization in the treatment of TBI, and we will focus on two novel antioxidants, mitoquinone (MitoQ) and edaravone. MitoQ can cross the blood brain barrier and cellular membranes to accumulate in the mitochondria and is thought to activate the Nrf2/ARE pathway leading to an increase in the expression of antioxidant enzymes. Edaravone is a free radical scavenger that leads to the mitigation of damage resulting from oxidative stress with a possible association to the activation of the Nrf2/ARE pathway as well.

## 1. Traumatic Brain Injury: Definition and Pathogenesis

Traumatic brain injury (TBI) is considered a major global health concern with no current FDA-approved drug for its treatment. TBI, defined as “an alteration of brain function, or an evidence of brain pathology, that is caused by an external force” has a major impact on the military personnel and the civilian population as well [1]. The incidence of TBI is estimated to be 939 in 100,000 worldwide with the major causes being falls, vehicle accidents, wars, and sports [2,3,4,5]. The mortality rate of TBI worldwide is estimated to be between 7% and 23% with 90% of TBI-related deaths occurring in developing countries [6,7]. Additionally, TBI imposes an economic burden on societies where its annual global cost reaches 400 billion dollars [8].

TBI is mainly classified according to severity into severe, moderate, and mild TBI, most commonly using the Glasgow Coma Scale (GCS) [9]. Mild TBI (mTBI), also known as concussion, affects about 740 per 100,000 people whereas severe TBI affects 73 per 100,000 people [2]. Post-concussive symptoms are regarded as transient and patients usually completely recover within three months [10]. Nevertheless, repetitive head traumas have been shown to have a grave long-term consequence such as the development of neurodegenerative diseases, including chronic traumatic encephalopathy (CTE) and Alzheimer’s diseases [11]. With the increasing worldwide attention towards sports, the increasing numbers of individuals enlisting in the military forces, and the overall increase in violence in societies, repetitive head trauma has resulted in as many as 1 million hospital admissions in 2018 [12].

The pathogenesis of TBI mainly comprises primary and secondary injuries. The primary injury is the direct result of the external physical force on the brain, whereas secondary injury happens minutes to days following the primary injury and consists of the molecular and chemical changes leading to neuronal damage [13]. However, the spatial separation between the primary and secondary injury is not clear in some forms of TBI, like repetitive mTBI, which shares some features with penetrating head TBI [14]. Ultimately, secondary damage can associate with behavioral, emotional, and cognitive deficits [15,16]. After the mechanical insult, there is a release of excitatory amino acids such as glutamate into the synapse that in turn overstimulate N-methyl-D-aspartate (NMDA) receptors. The resultant is an overload of Ca^2+^, along with increased depolarization due to ionic imbalance [17,18,19]. High levels of Ca^2+^ can cause an intracellular Ca^2+^-dependent Ca^2+^ release and subsequently activate Ca^2+^-dependent enzymes including proteases, lipases, and endonucleases that can eventually lead to protein degradation, disruption of protein phosphorylation, and protein aggregation like tau proteins [17]. The excess of intracellular Ca^2+^ and excitotoxicity lead to the excessive production of reactive oxygen species (ROS) and ultimately to oxidative stress. This primarily occurs in mitochondria where the increased Ca^2+^ stimulates ROS production through membrane transition pore (MTP) activation, cytochrome *c* (cyt *c*) release, and respiratory chain inhibition [20]. In addition, secondary injury post TBI also encompasses neuro-inflammation, blood brain barrier (BBB) dysfunction, axonal injury and metabolic disturbance.

## 2. Oxidative Stress in TBI

Oxidative stress represents a state where oxygen levels along with oxygen-derived free radicals overwhelm the scavenging antioxidant system. These include agents like hydrogen peroxide (H_2_O_2_), superoxide anions (O_2_^−^), hydroxyl (OH¯), and peroxyl (ROO¯) radicals [21].

Once injury-induced excitotoxicity occurs, the excess of Ca^2+^ promotes the production of ROS as well as nitric oxide (NO) where protective mechanisms such as antioxidants fail to control these radicals, leading to oxidative stress [22]. Increased concentrations of free radicals result in the alteration of various macromolecules including DNA, proteins, and lipids which eventually impairs various cellular processes [23]. The reversible and irreversible alterations of those macromolecules predispose individuals to a wide range of disorders including neurodegenerative diseases [24,25].

Mitochondria act as both the source and the target of free-radical oxidation. Unlike nuclear DNA, mitochondrial DNA has no nucleotide-excision repair pathways and is not protected by histones. Thus, mitochondrial DNA becomes particularly prone to mutations. Mutated mitochondrial DNA causes a bioenergetic deficit where adenosine triphosphate (ATP) production markedly decreases and free radical production significantly increases [26]. Brain tissue is specifically vulnerable to oxidative damage due to its high oxidative metabolic activity, relatively low antioxidant capacity, and low repair mechanisms [27,28]. In TBI, ROS can be produced via the arachidonic acid (AA) cascade activity, mitochondrial leakage, catecholamine oxidation, and by neutrophils [29,30]. In addition, NADPH oxidases (Nox) are a family of membrane enzymes that reduce oxygen into ROS. Nox play a major role in the pathophysiology of the nervous system and they have a crucial contribution in the development of secondary injury after TBI. It was shown that the activity of Nox is elevated 1 h after TBI and is set in action by microglia and that the inhibition of Nox can attenuate the secondary injury post TBI [31,32]. Hence, Nox inhibition can also act as a therapeutic target [33].

**Figure 1:**
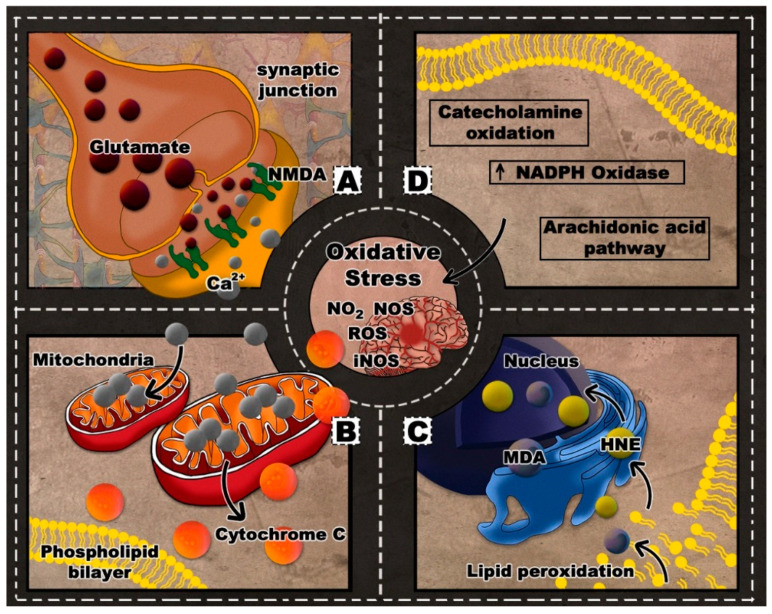
Oxidative stress in traumatic brain injury (TBI): (**A**) excitotoxicity; (**B**) Ca^2+^ sequestration and cyt *c* release; (**C**) lipid peroxidation; (**D**) oxidative stress pathways.

The severity of injury in TBI can be correlated with the degree of ROS-related tissue damage [23] and mitochondrial dysfunction [34]. Oxidative stress in repetitive TBI is prominently manifested as lipid peroxidation (LP) of neuronal, glial, and vascular cell membranes as well as myelin [35]. LP can result in the breakdown of polyunsaturated fatty acids in lipid membranes, further disrupting the ionic gradients and potentially leading to membrane lysis [29]. In an attempt to restore the ionic balance, ionic pumps along the membrane are activated. Hence, more glucose would be consumed, energy stores become increasingly depleted, and the mitochondrial Ca^2+^ influx would increase. Such impaired oxidative metabolism and glycolysis can lead to lactate production, acidosis, and edema [29,30,36]. The process of sequestering Ca^2+^ in the mitochondria could lead to cell death either directly by apoptosis or indirectly through the loss of oxidative phosphorylation and failed production of ATP. More specifically, Ca^2+^ overload could play a leading role in the mitochondrial cyt *c* release, caspase activation, and apoptosis [21,36]. Figure 1 depicts a summary of the oxidative stress pathology in the context of TBI.

To date, no treatment has been effective in eradicating the consequences of injury. Instead, therapeutics have focused on alleviating the impact of secondary injury and managing its biochemical contributors. That being said, and with a growing body of evidence on the role of oxidative stress in TBI, antioxidants are being considered as potential therapeutics [22,23,35,37].

## 3. Therapeutic Options Targeting Oxidative Stress in TBI

The mechanisms involved in oxidative damage and LP provide potential targets for their inhibition. In this review, we will briefly discuss the history of some discovered compounds that have a direct and indirect antioxidant activity in TBI, followed by an elaborate discussion regarding the two novel promising therapeutics, mitoquinone (MitoQ) and edaravone.

### 3.1. A Brief History of Multiple Antioxidants Utilized in TBI

The different mechanisms involved in oxidative stress can be considered as putative targets for the treatment of TBI. For example, there are compounds involved in inhibiting LP initiation by preventing the formation of ROS and reactive nitrogen species (RNS) such as nitric oxide synthase (NOS) inhibitors [35,38,39,40]. Another approach is to inhibit enzymes of the AA cascade, like cyclooxygenase and 5-lipoxygenases, which eventually blocks the formation of O_2_¯. Cyclooxygenase inhibition by nonsteroidal anti-inflammatory agents like ibuprofen were found to be neuroprotective in TBI models [35,39,40,41].

A second indirect approach to inhibit LP initiation involves scavenging radical species like O_2_¯, OH¯, NO_2_, and CO_3_^−^ to prevent their interaction with fatty acids. For example, the superoxide dismutase (SOD) enzyme was found to scavenge O_2_¯ [35,39,40,42] and nitroxide antioxidant (tempol) was shown to scavenge NO_2_ and CO_3_^−^ [35,39,40,43]. The 21-aminosteroid LP inhibitor tirilazad (also known as U74006F) was used in several animal studies and human trials and was found to inhibit free radical-induced LP by scavenging lipid Peroxyl radicals (LOO^−^) and to stabilize membranes, which limits the interaction between an LOO^–^ and adjacent fatty acids [44,45,46,47].

The third category of indirect-acting antioxidants involves preventing the “chain reaction” propagation of LP once it has begun by scavenging LOO^−^ or alkoxyl (LO^−^) radicals. An endogenous example of such scavengers is vitamin E (Vit E). Vit E can only quench one LOO^−^ and cannot scavenge another LOO^−^ until it is reduced back to its active form by receiving an electron from other endogenous antioxidants such as ascorbic acid (Vit C) or glutathione. This is known as the tripartite antioxidant system [35,39,40,48]. Other LOO^−^ scavengers include curcumin [49], resveratrol, melatonin [50], and lipoic acid [51]. However, the most potent LOO^−^ scavenging-LP inhibitor discovered is the 2-methylaminochroman compound, also known as U-83836E. The latter combines the LOO^−^ scavenging antioxidant chroman ring structure of Vit E with the bis-pyrrolopyrimidine moiety of tirilazad [52]. A second approach to inhibit the propagation of LP reactions is by chelating free iron, ferrous (Fe^2+^) and ferric (Fe^3+^), which can catalyze the breakdown of lipid hydroperoxides (LOOH). The prototypical iron-chelating drug is the bacterially-derived tri-hydroxamic acid compound, deferoxamine [53,54,55].

However, most of these approaches were not recognized as fully efficient or potent for various reasons. Some have the potential to interfere with the physiological state like the NOS inhibitors. Others are only efficient in cases of minor oxidative stress like the tripartite antioxidant system which are also rapidly consumed during the early minutes and hours after TBI. Some have a short therapeutic window and would have to be administered promptly in order to be able to interfere with the initial peak of post-traumatic free radical production like the radical scavenger SOD and the nitroxide antioxidant tempol. Moreover, many compounds failed to show benefits in clinical trials due to multiple preclinical inadequacies like dose-response relationships, pharmacokinetic-pharmacodynamic correlations, therapeutic window, and optimal dosing regimen and treatment duration [35]. Recently, two antioxidants, edaravone and MitoQ, have raised interest.

### 3.2. Edaravone

Edaravone is an antioxidant that has been used in Japan since 2001 in the management of neurological symptoms and functional disorders associated with acute ischemic stroke. The drug scavenges free radical post-ischemic events, thereby mitigating oxidative injury in neurons. Besides its anti-oxidative property, it has been shown to play a role in decreasing nitric oxide production, matrix metalloproteinases activity, inflammation, and apoptotic cell death, thus fitting into the class of multi-target compound [56]. Due to its promising effects, edaravone was launched as a therapeutic drug for amyotrophic lateral sclerosis (ALS) in Japan and Korea in 2015, and was later approved by U.S. FDA in 2017 and in Canada in 2018 [57].

#### 3.2.1. Edaravone in ALS

The exact mechanism of action by which edaravone exerts its therapeutic effects in ALS is unknown; however, it may be due to its anti-oxidative property since oxidative stress is a part of the cascade leading to motor neuron death in patients with ALS [58]. In multiple in-vivo studies on ALS, edaravone was capable of suppressing the nitration of tyrosine residues, attenuating motor decline and muscle weakness, reducing the abnormal disposition of SOD1 in the spinal cord, preserving motor neurons, decreasing denervation atrophy, and decreasing motor neuron degeneration [59,60,61]. In vitro studies found that edaravone ameliorated the harmful effects of neurotoxins and oxidants in cultured neuronal cells by reducing ROS generation, decreasing cytotoxicity, and increasing cell survival. The mechanism underlying these neuroprotective effects was associated with the nuclear factor erythroid 2-related factor 2/anti-oxidant responsive element (Nrf2/ARE) pathway in which increased Nrf2 expression and translocation from the cytoplasm to the nucleus increased the expression of the antioxidant enzymes SOD and heme oxygenase-1 (HO-1) [62,63]. The effects of edaravone on mitochondrial dysfunction are poorly addressed; however, it was shown that edaravone improves mitochondrial functions and modulates mitochondrial-dependent apoptosis pathways by preventing cyt *c* release and inhibiting caspase-3 activation [64].

#### 3.2.2. Edaravone in TBI

**Figure 2:**
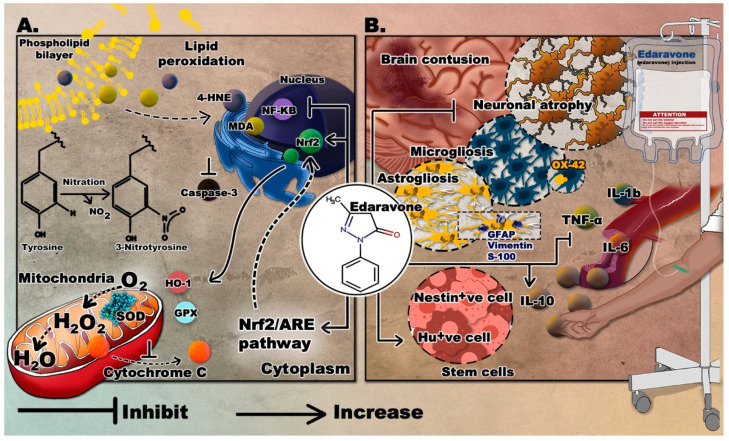
The proposed neurotherapeutic mechanisms of edaravone. (**A**) Suppression of oxidative stress by edaravone; (**B**) effects of edaravone in TBI. Molecular graphics of the superoxide dismutase (SOD) enzyme was visualized using the 3D Protein Imager online server [71].

For the past decade, extensive research focused on the therapeutic effects of edaravone in several neurological disorders, including TBI, where it was shown to be safe and efficient. The novelty of this drug relies in its multi-target mechanism which makes it a drug of choice compared other antioxidants. The findings from several animal studies are summarized in Table 1 and the different outcomes of edaravone are displayed in Figure 2.

##### Dose, Therapeutic Window, and Time Frame

In studies on rodent models of TBI, edaravone was given via the intravenous route at a dose of 1.5 or 3 mg/kg [65,66]. The effect of edaravone in rodents was studied at acute and subacute time points (1/3 and 7 days) post injury [67,68]. Regarding the therapeutic time window, a study found that edaravone can exert its effect when administered up to six hours after injury [65]. On the other hand, in a study on closed head injury, edaravone only exerted a significant effect when it was administered 1 h after injury, and the effect was dose-dependent [69].

##### Providing Neuroprotection

Studies on rodent models of TBI showed that edaravone can significantly reduce the injury or lesion volume [65,66,70] and can increase the number of Nissl-positive neurons in the injured area and hippocampus [66,70]. Moreover, apoptosis, as evident by the apoptotic index (TUNEL), was significantly decreased in the edaravone group at 24 h post injury [70]. Other DNA damage and apoptosis markers, like single stranded DNA (ssDNA) and 8-hydroxy-20-deoxyguanosine (8-OHdG) were also decreased in the injured brain area at acute and sub-acute time points after TBI [67,68]. Moreover, limited irregular nuclear membranes, lack of perinuclear edema, and absence of degeneration were observed in small-sized myelinated axons and non-myelinated axons in the edaravone treated group as compared to extensive irregularity, margination and clumping of neurons in the TBI group. Edaravone is also capable of attenuating caspase-3 apoptotic activity at the impact area [66].

##### Antagonizing Oxidative Stress

An important role has been attributed to edaravone in fighting oxidative stress in TBI. In a mouse model of severe TBI, the total volume of nitrotyrosine (NT) and the production of ROS were suppressed following edaravone administration. Additionally, the expression of Nrf2, a transcription factor, and its downstream genes were elevated by edaravone [65]. Similarly, in a mouse model of concussion, NT levels were decreased in the cortex and hippocampus when edaravone was administered [69]. In one study, H_2_O_2_ level decreased, and SOD and GPx increased following edaravone treatment [70]. In another study, edaravone treatment notably attenuated oxidative stress as evident by the decrease in malondialdehyde (MDA), a reactive aldehyde formed from LP, the decrease in nitrous oxide (NO) levels, the reduction in the activity of inducible nitric oxide synthase (iNOS), an enzyme mainly expressed in macrophages to produce NO, and the increase in the activity of SOD [66]. Moreover, at acute and subacute time points following TBI, in the edaravone treated group, 4-hydroxy-2-nonenal (4-HNE, another lipid peroxidation marker) immunopositive cells were scarcely present around the injured brain area [67,68]. Similarly, in a mouse model of concussion, 4-HNE was significantly suppressed in the cortex and hippocampus 1 day after injury, when edaravone was administered immediately after concussion [69].

##### Attenuating the Immune Response and Managing Cerebral Edema

Several studies investigated the effect of edaravone on the inflammatory response, including the involved immune cells and cytokines. The production of the inflammatory cytokines, tumor necrosis factor α (TNFα), and interleukins 6 and 1-b (IL-6, IL-1b) were found to be significantly attenuated in brain tissue, whereas IL-10, an anti-inflammatory cytokine, was increased, following edaravone administration. Besides, a reduced expression of nuclear factor kappa B (NF-κB) was detected compared with untreated controls. There was also a negative correlation between the inflammatory cytokines and SOD, and a positive correlation with MDA, suggesting a link between edaravone’s effect on oxidative stress and neuroinflammation [66,70]. Edaravone is also capable of suppressing astrogliosis and microgliosis where cells expressing markers of astrocyte activation (GFAP, Vimentin, and S-100) and cells expressing markers of microglia activation (OX42) were greatly reduced in the brain tissue surrounding the cortical contusion following edaravone administration [66]. One study showed that edaravone was capable of decreasing cerebral edema and blood brain barrier (BBB) permeability, in a dose-dependent manner, as measured by water content and Evans blue dye diffusion [66].

##### Enhancing Stem Cell Production

Some studies have reported a role of edaravone in the production of stem cells. A significant increase in nestin (neural precursor marker) positive cells was noted around the injured brain area in edaravone treated group at 3 and 7 days after TBI. Similarly, the number of isolated and cultured spheres was significantly increased as compared to the saline vehicle group [67]. In addition, there was a significant increase in the number of Hu-positive cells (neuronal cell marker) at 7 days following TBI in the edaravone treated group [68].

##### Modifying Behavior

The role of edaravone in modulating the behavioral response in rodents has also been demonstrated by multiple studies. The neurological severity score (NSS) of mice in an untreated TBI group was significantly higher compared to the edaravone treated group [70]. In addition, the performance of injured rats in beam-balancing and prehensile traction tests was enhanced by edaravone. Such improvement was correlated with the number of hippocampal CA3 neurons [66]. As for learning and memory, the arrival time to platform, in Morris water maze, was significantly decreased in the Edaravone group [68]. Similarly, in a mouse model of brain concussion, depressive-like behavior was prevented by edaravone, as evident by the decrease in immobility time in the forced swim test (FST). The latter was also negatively correlated with the dose of edaravone administered [69].

##### Use of Edaravone in Patients with TBI

To test the efficacy of edaravone, numerous clinical trials were carried out in ALS patients [72,73,74] and in stroke patients [75,76,77]; however, its overall safety and efficacy in patients with TBI is still uncertain. In fact, studies on edaravone in the clinical setting are still scarce. The first clinical study to assess the efficacy of edaravone was conducted in 2006 on 17 patients with TBI where alkoxyl radicals (OR^−^) quantification was used as an endpoint. High levels of OR^−^ were observed in the blood samples of all patients and were significantly reduced at 20 min after the intravenous administration of 30 mg of edaravone. On the other hand, OR^−^ levels in the 4 patients who did not receive edaravone remained unchanged [78].

### 3.3. Mitoquinone (MitoQ)

**Figure 3:**
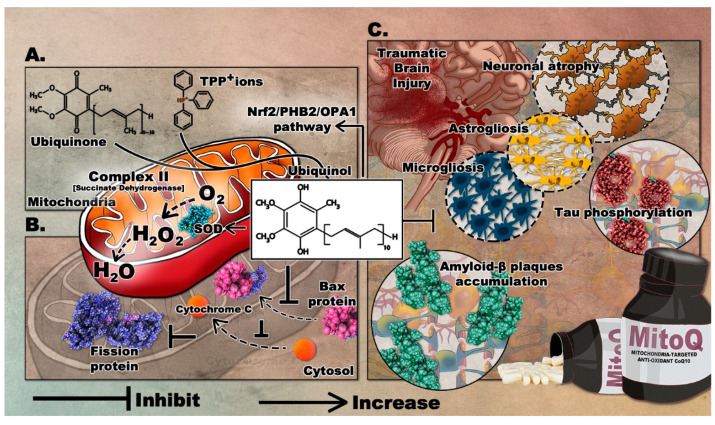
The mediated therapeutic mechanisms of MitoQ targeting the mitochondria with its proposed implications on TBI. (**A**) Formation of MitoQ; (**B**) role of MitoQ in attenuation of neuronal apoptosis; (**C**) inhibitory role of MitoQ following TBI. Molecular graphics of the Superoxide Dismutase (SOD) enzyme, fission protein, Bax protein, Amyloid-ß plaques, and phosphorylated Tau were visualized using the 3D Protein Imager online server [71].

MitoQ is among the widely used antioxidants that target the mitochondria. It was developed in the 1990s to readily penetrate the BBB and neuronal membranes, where it is concentrated into several hundred-folds within the mitochondria where it mediates the local anti-oxidative capacity [79]. MitoQ is formed by covalently binding ubiquinone or coenzyme Q, an endogenous antioxidant and a component of the mitochondrial electron transport chain (ETC), to triphenylphosphonium (TPP^+^) ions. TPP^+^ is a lipophilic cation that drives the ubiquinone moiety into the inner mitochondrial membrane, by the negative electrochemical potential (see Supplementary Figure S1) [80]. Within the ETC, complex II, also known as succinate dehydrogenase, reduces MitoQ ubiquinone moiety to the active antioxidant ubiquinol which scavenges excess ROS. After reducing ROS, ubiquinol is oxidized to ubiquinone and then recycled by complex II. MitoQ is a poor substrate for complexes I and III, so it cannot substitute for endogenous ubiquinone and therefore does not take part in the mitochondrial respiration; thus acting as a renewable antioxidant [81]. Notably, studies have shown that MitoQ produces ROS during its redox cycling [82]. This perturbation may trigger defense cascades to protect cells. MitoQ possibly induces the oxidation of Keap1 and its subsequent degradation and release of Nrf2 [83]. This eventually leads to the upregulated expression of antioxidant enzyme genes as mentioned previously. The formulation of MitoQ is summarized in Figure 3A.

#### 3.3.1. Effects of MitoQ in Preclinical Studies of Neurodegenerative Diseases

In animal and in vitro models of PD, MitoQ has demonstrated positive outcomes. When used in SH-SY5Y cell line, MitoQ reduced 6-OHDA-induced mitochondrial fragmentation. It inhibited mitochondrial fission protein and the translocation of pro-apoptotic protein (Bax) in the mitochondria [84]. In another study, neuroprotective effects were demonstrated in both cellular and mouse models of PD, in which MitoQ treatment inhibited the loss of dopaminergic neurons and enhanced behavioral performance [85]. Furthermore, a recent study performed on a Zebrafish PD model showed that MitoQ could improve the oxidant-antioxidant balance, ameliorate the expressions of PD- related genes, and enhance the overall mitochondrial function [86].

Moreover, in a Huntington’s disease (HD) mouse model, MitoQ treatment enhanced the fine motor control and reduced markers of oxidative damage in muscles. It also attenuated overactive autophagy-induction associated with muscle wasting [87]. Such intervention improved muscle performance and protected from proteostasis impairment, suggesting that mitochondrial-targeted antioxidants may have promising therapeutic effects in neuromuscular disorders.

Furthermore, in intracerebral hemorrhagic (ICH) mice, MitoQ reduced white matter injury, improved neurological performance, and decreased motor-evoked potential latency [88]. Such effects may be due to the reduced oligodendrocyte death and demyelination and inhibition of mitochondrial injury.

Due to its promising effects in neuromuscular disorders, MitoQ was studied in several ALS-models. In transgenic ALS mice carrying the G93A mutated human SOD1, MitoQ treatment improved mitochondrial function in both the spinal cord and the quadriceps muscle [89]. Interestingly, the nitro-oxidative markers in the spinal cord of treated animals were significantly reduced, coupled with a recovery of neuromuscular junctions and an enhancement in hind-limb strength. MitoQ treatment also significantly prolonged the life span of SOD1 (G93A) mice.

In an Alzheimer’s disease (AD) mouse model, MitoQ treatment significantly improved spatial memory retention and reduced brain oxidative stress, astrogliosis, microglia cell proliferation, tau hyper-phosphorylation, and amyloid plaque formation [90]. MitoQ treatment also extended the lifespan of 3xTg-AD mice, thus emphasizing its role in dampening the neuropathology responsible for the accelerated death rate of these mice.

The probable molecular mechanism underlying the neuroprotective effects of MitoQ was addressed in a recent in-vivo study. In a subarachnoid hemorrhage (SAH) rat model, treated animals showed refined mitochondrial morphology, decreased BBB disruption, and improved neurological performance. These were attributed to the Nrf2/PHB2/OPA1 pathway where MitoQ could elevate Nrf2 levels by binding to its antagonist protein Keap1 [91]. A summary of findings in animal models of neurodegenerative disease following the administration of MitoQ is presented in Table 2.

#### 3.3.2. Effects of MitoQ in Preclinical Models of TBI

Evaluating the effects of MitoQ in a TBI model showed significant improvements in behavioral and molecular outcomes. Considering the fact that mitochondria play a crucial role in the pathogenesis of TBI, delivering a mitochondria-targeted antioxidant protects mitochondria against oxidative stress thereby preventing neuronal death. As discussed, mitochondria are a major source of ROS and hence are particularly sensitive to oxidative injury which further exacerbate ROS production. This can lead to a vicious cycle of an increasing level of mitochondrial injury and eventually apoptotic cell death and metabolic imbalance [94]. Accordingly, available research data indicate that decreased mitochondrial oxidative stress can suppress or delay the progression of TBI [95].

The first study to demonstrate the effects of MitoQ in TBI was recently completed. The treatment was shown to significantly improve neurological deficits, alleviate brain edema, and inhibit cortical neuronal apoptosis in a TBI mouse model [92]. Mice treated with 4 mg/kg of MitoQ showed significantly improved neurobehavioral functions, coupled with increased activity of different antioxidant enzymes, including SOD and GPx. The effect of MitoQ on TBI-induced apoptosis was evaluated by quantifying the expression of the mitochondrial apoptosis-related proteins. Bax protein translocation to the mitochondria and the cytosolic release of cyt-*c* were reduced, confirming that MitoQ can attenuate neuronal apoptosis in the cortical contusion post-injury. Remarkably, MitoQ accelerated the Nrf2 nuclear translocation and subsequently upregulated the expression of downstream proteins, including HO-1 and quinone oxidoreductase 1. Therefore, such findings demonstrate that MitoQ mediates its neuroprotective effects via activating the Nrf2/ARE pathway. The role of mitoQ in apoptosis is depicted in Figure 3B.

Considering its potential effects in preclinical studies of several neurological diseases, MitoQ intervention can be a promising drug to attenuate the progression of TBI, especially that oxidative stress and mitochondrial impairment play a significant role in the pathogenesis of TBI. The proposed role of MitoQ in TBI can be found in Figure 3C.

#### 3.3.3. Safety of MitoQ in Clinical Trials in Neurodegenerative Disorders

A phase II randomized double-blind clinical study (NCT00329056) was conducted on 128 newly diagnosed untreated patients with Parkinson’s disease (PD). Patients were given a daily oral dose of 40 or 80 mg of MitoQ for 12 months and were compared with patients that received a placebo. The trial was verified in 2010 and the results were negative, showing no significant improvement in patients with PD [96]. The study argued that this finding should be taken into account when considering the oxidative stress hypothesis for the pathogenesis of PD. The trial, however, demonstrated that it is safe to target human mitochondria with antioxidants over a prolonged period and the efficacy of the treatment might be dependent on the time course of the disease.

Recently, another study (NCT03514875) has been initiated to evaluate the effects of MitoQ on the blood flow of the carotid artery in patients with mild cognitive impairment. The targeted outcome measures include oxidative stress, cerebrovascular oxygenation, brain electrical activity, and endothelial function. It is a randomized, double blind, crossover clinical trial involving 12 participants (still recruiting) that is estimated to be completed in 2021.

## 4. Conclusions

Antioxidants such as MitoQ and edaravone were shown to have a neuroprotective effect in several pre-clinical studies. Such outcomes include enhanced neurological and motor functions, as well as reduced oxidative stress, neuroinflammation, and apoptosis; i.e., alleviation of TBI hallmarks. Treating TBI remains a necessity considering its growing impact on societies. Despite the potential role of antioxidants, further studies are needed to elucidate the mechanisms by which antioxidants work and to find ways to enhance their mode of delivery. Furthermore, clinical studies are also needed to prove the efficacy of antioxidants as they move from pre-clinical settings to bedside. Hence, drug repurposing could be a promising initiative in managing TBI.

## Figures and Tables

**Table 1:** The use of edaravone in animal models of TBI.

Animal Species	TBI Model/Device	Drug Administration	Behavioral Outcomes Following Edaravone Administration	Molecular Outcomes Following Edaravone Administration	Ref
**C57BL/6 mice**	Electromagnetic CCI	Injection of 3 mg/kg (100–150 µL volume) into jugular vein	-	- Decrease in NT production - Decrease in O_2_¯generation - Increase in NRF2 production	[65]
**Male Sprague-Dawley rats**	Feeney’s weight-drop model	Injection of 1.5 mg/kg via vena caudalis at 2 h and 10 h after TBI	-	- Increase in Nissl-positive neurons in the injured area and hippocampus - Reduced lesion volume - Attenuated caspase-3 activity - Decrease in MDA, NO and iNOS - Increase in SOD production - Decrease in GFAP, Vimentin, S-100, and OX42 expression - Decrease in Evans blue diffusion	[66]
**10-week old male Wistar rats**	Pneumatic-controlled injury device	Intravenous injection of 3 mg/mL edaravone at 3 mg/kg directly after TBI	Decrease in the arrival time to platform (MWM)	- Increase in the number of Hu-positive cells - Attenuation of ssDNA and 8-OHdG in injured area - Decrease in 4-HNE positive cells in injured area - Increase in nestin positive cell and number of spheres in injured area	[67,68]
**dMale C57BL/6 mice (12–16 weeks old)**	Concussive head trauma device (using a vertical metal guide tube)	Intravenous injection of 3 mg/mL edaravone at 3 mg/kg, directly after TBI	Decrease in immobility time in FST	- Decrease in NT levels in the cortex and hippocampus - Suppression of 4-HNE in the cortex and hippocampus	[69]
**C57BL/6 mice**	Pneumatic controlled cortical impact	Intraperitoneal injection of 3 mg/kg of edaravone, 1 h post-TBI	Decrease in neurological deficits (NSS)	- Decrease in H_2_O_2_ levels - Increase in SOD and GPx production - Decrease in lesion volume - Increase in Nissl-positive neurons in the injured area and hippocampus - Decrease in apoptotic index (TUNEL)	[70]

**Table 2:** The neuroprotective effects of MitoQ in mice models of neurodegenerative diseases.

Model	Experimental Procedure	Behavioral Outcomes	Molecular Outcomes	Biomarkers	Ref
**Huntington’s disease**	- R6/2 mice as HD model - 500 μM of MitoQ administered ad libitum in drinking water, starting 5 weeks of age - Drug renewal twice a week until the end of the experiments (at 11 weeks of age)	- Enhancement of fine motor control in open field test - Improved performance in the vertical pole test	- Reduced oxidative stress in muscles - Decreased protein ubiquitination and autophagy induction in muscle	- Reduction of SOD2 levels in muscle - Decreased LC3-II	[87]
**TBI**	- Single impact onto the left frontal bone using 200 g weight-drop - 4 mg/kg MitoQ injected peritoneally, 30 min after TBI - Neurobehavior assessed at 1,3 and 7 days after TBI - Molecular assessment done 24 h post-TBI	Lowered NSS scoring at day 1 and 3 post-TBI	- Attenuated brain water content - Ameliorated neuronal apoptosis in the cortex - Regulated expression of mitochondrial apoptosis-related proteins	- Increased levels of SOD and GPx levels - Reduced TUNEL-positive cells - Increased expression of cytosolic Bax protein compared to the mitochondria and increased expression of mitochondrial cyt *c* compared to cytosol - Elevated expression of nuclear Nrf2 and increased expression of downstream genes: HO-1 and NQO-1	[92]
**ALS**	- Transgenic ALS mice carrying the G93A mutated human SOD1, strain B6SJL-TgN(SOD1-G93A)1Gur - 500 μM of MitoQ administered in drinking water from 90 days of age until death	- Increased mean survival in males and females - Improved grip-strength performance at the end stage of the disease only in female	- Attenuated nitroxidative stress - Reduced motor neuron loss and astrogliosis in the lumbar spinal cord	- Reduced number of glial-like HO-1-immunoreactive cells per area in the spinal cord of SOD1G93A mice - Reduction (54%) in GFAP content in the spinal cord from MitoQ-treated SOD1G93A mice vs. the vehicle- treated	[89]
**Alzheimer’s disease**	- 3xTg-AD mouse model expressing three mutant human genes: amyloid precursor protein, APP_swe_; presenilin-1, PS1_M146V_; four-repeat tau. tau_p301L_ - Starting 2 months of age, mice given 100 µM of mitoQ in drinking water for 5 months	Enhanced learning and spatial memory retention by MWM	- Reduced oxidative stress - Protected against lipid peroxidation - Decreased synaptic loss - Decreased Aβ accumulation	- Enhanced GSH/GSSG ratios in the brain - Decreased loss of synaptophysin	[93]
**Parkinson’s Disease**	- MPTP (25 mg/kg) injected intra-peritoneally via a single dose daily starting day 2 for 5 days - 4 mg/kg of MitoQ administered by oral gavage for 1 day (before MPTP), 5 days (with MPTP), and 7 days (post MPTP)	Improved locomotor activities in open field and Rotarod	- Attenuated depletion of dopamine and its metabolites induced by MPTP toxicity - Protection of nigrostriatal axis	- Less decrease in dopamine levels - Restoration of DOPAC and HVA levels - Less reduction of TH-positive fibers and neurons in striatum and substantia nigra	[85]

## Supplementary Materials

The following are available online at https://www.mdpi.com/2076-3921/9/10/943/s1, Figure S1: Mitiquinone (MitoQ) structure. MitoQ incorporates a triphenylphosphonium cation linking a ubiquinone moiety via an aliphatic 10-carbon chain. MitoQ is accumulated in the inner mitochondrion membrane at a 5–20 folds, facilitated by the lipophilic cationic triphenylphosphonium (TPP^+^).
