# Supplementary material for: Traumatic Brain Injury: Oxidative Stress and Novel Anti-Oxidants Such as Mitoquinone and Edaravone
Source: Antioxidants (Basel). 2020 Oct 1;9(10):943. doi: 10.3390/antiox9100943 (PMC7601591; doi:10.3390/antiox9100943)
Supplement: Supplementary file 1 [file antioxidants-09-00943-s001.zip › antioxidants-884937-supplementary.docx]

Supplementary materials


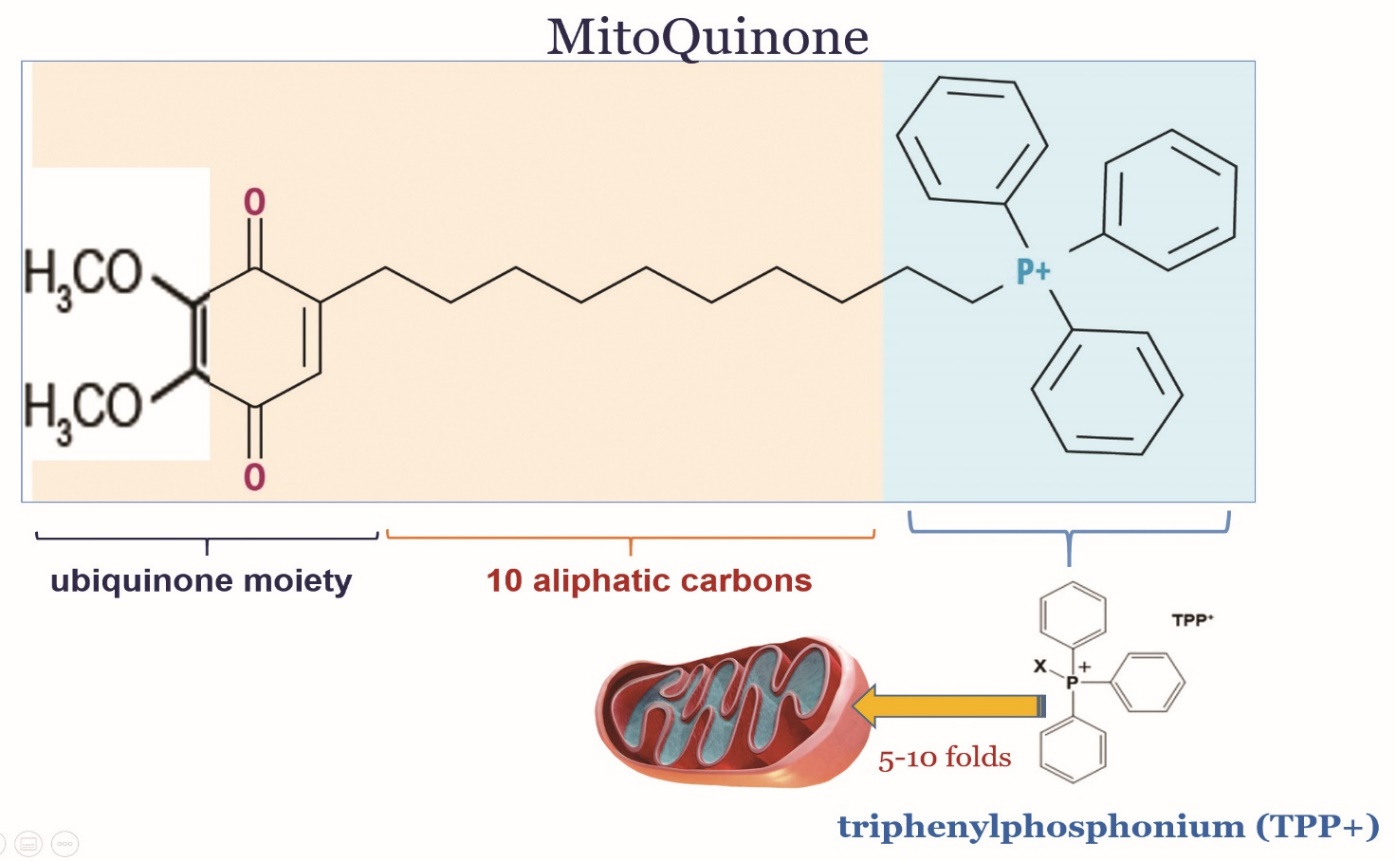


**Figure S1**. Mitiquinone (MitoQ) structure. MitoQ incorporates a triphenylphosphonium cation linking a ubiquinone moiety via an aliphatic 10-carbon chain. MitoQ is accumulated in the inner mitochondrion membrane at a 5–20 folds, facilitated by the lipophilic cationic triphenylphosphonium (TPP^+^).
